# Cartilage Dysfunction in ALS Patients as Side Effect of Motion Loss: 3D Mechano-Electrochemical Computational Model

**DOI:** 10.1155/2014/179070

**Published:** 2014-06-03

**Authors:** Sara Manzano, Eamonn A. Gaffney, Manuel Doblaré, Mohamed Hamdy Doweidar

**Affiliations:** ^1^Group of Structural Mechanics and Materials Modelling (GEMM), Aragón Institute of Engineering Research (I3A), University of Zaragoza, 50018 Zaragoza, Spain; ^2^Mechanical Engineering Department, School of Engineering and Architecture (EINA), University of Zaragoza, María de Luna s/n, Betancourt Building, 50018 Zaragoza, Spain; ^3^Biomedical Research Networking Center in Bioengineering, Biomaterials and Nanomedicine (CIBER-BBN), 50018 Zaragoza, Spain; ^4^Wolfson Centre for Mathematical Biology (WCMB), Mathematical Institute, Oxford University, Oxford OX1 2JD, UK

## Abstract

Amyotrophic lateral sclerosis (ALS) is a debilitating motor neuron disease characterized by progressive weakness, muscle atrophy, and fasciculation. This fact results in a continuous degeneration and dysfunction of articular soft tissues. Specifically, cartilage is an avascular and nonneural connective tissue that allows smooth motion in diarthrodial joints. Due to the avascular nature of cartilage tissue, cells nutrition and by-product exchange are intermittently occurring during joint motions. Reduced mobility results in a change of proteoglycan density, osmotic pressure, and permeability of the tissue. This work aims to demonstrate the abnormal cartilage deformation in progressive immobilized articular cartilage for ALS patients. For this aim a novel 3D mechano-electrochemical model based on the triphasic theory for charged hydrated soft tissues is developed. ALS patient parameters such as tissue porosity, osmotic coefficient, and fixed anions were incorporated. Considering different mobility reduction of each phase of the disease, results predicted the degree of tissue degeneration and the reduction of its capacity for deformation. The present model can be a useful tool to predict the evolution of joints in ALS patients and the necessity of including specific cartilage protectors, drugs, or maintenance physical activities as part of the symptomatic treatment in amyotrophic lateral sclerosis.

## 1. Introduction

Amyotrophic lateral sclerosis (ALS) is a neurodegenerative disorder characterized by progressive loss of motor units leading to muscle atrophy, weakness, and immobilization [1]. Despite substantial effort to decipher the etiology of the disease, the primary events triggering the pathology remain to be elucidated. Currently, there is no cure for the disease and its management is focused on symptomatic treatment and nutritional supply to extend the life expectancy, improve quality of life, and help to maintain the patient's autonomy for as long as possible [2].

Important long term problems are facing ALS patients, such as osteoarthritis and cartilage progressive degeneration as a consequence of the chronic reduction in joints mobility [3, 4].

**Figure 1:**
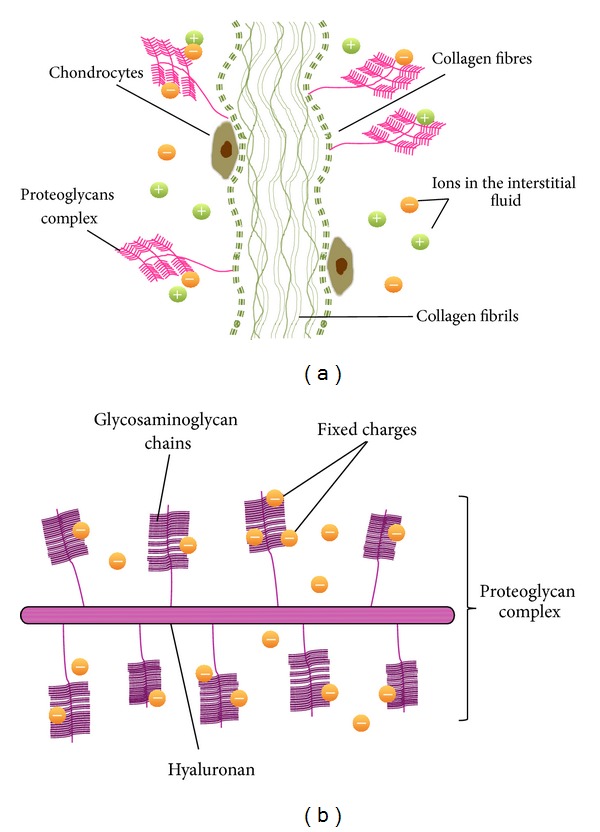
Schematic illustration of articular cartilage microstructure and its main components. (a) Constituents of the cartilage tissue: chondrocytes, proteoglycans complex, collagen fibers, and ions dissolved in the interstitial fluid. (b) Proteoglycan macromolecule aggregate with charged-glycosaminoglycan side chains attached to a hyaluronic chain.

Due to the avascular nature of cartilage, cells nutrition and by-product exchange are diffusive mediated processes occurring intermittently in the articular movements [5]. In case of ALS patients, due to their inability to exercise and lack of continuous motion, these diffusive processes are ceased. However, no studies have looked into the correlation between progressive loss of motion and cartilage degeneration in ALS patients. Such studies would help clinicians to determine the need of specific cartilage treatments. Cartilage tissue is mainly composed of fibrous network of proteins such as collagen fibers, proteoglycans (PGs), and elastin. This network is surrounded by synovial which is fluid mainly composed of water and ions (Na^+^ and Cl^−^ among others) (Figure 1(a)). Since cells occupy a small fraction of the total volume of cartilage, transport of fluid and solute substances mainly depends on the properties of the extracellular matrix (ECM) [6].

Substantial numerical simulations have emerged to reproduce* in vivo* cartilage behavior [7–10]. However, most of these models do not include ions role among the main components of the tissue, which is a key aspect when simulating cartilage degenerative diseases [11].

In this sense, capturing the influence of each component (ECM, water, and ions) within the macroscale behavior (deformation as a consequence of ion and water fluxes) requires the consideration of the cartilage as a multiphasic poroelastic material with three main components; the liquid phase (water), the ion phase (Na^+^ and Cl^−^ ions), and the solid phase, collagen fibers, PGs, and proteins [12–14]. Attached to PGs there are glycosaminoglycan chains (GAGs) which contain sulphate and carboxyl groups. PGs become negatively charged in the physiological environment through interaction with anions (Cl^−^). These ECM-attached anions, so-called “fixed charge density” (FCD), present a coupled movement with collagen fibers given rise to a swelling pressure in the tissue (Figure 1(b)).

Under normal physiological conditions, the fixed negative charges in ECM are electrically neutralized by mobile cations contained in the interstitial fluid [15]. The difference in ion concentration between the ECM and interstitial fluid generates an osmotic pressure (Donnan osmotic pressure) leading to the typical cartilage swelling and shrinking phenomena [16, 17]. These phenomena play a significant role in cartilage behavior, flexibility, and stability required to support loads and resist stretching, since they are responsible for the fluid and ion content within the tissue [18]. In ALS patients, where the mobility is reduced, the normal balance of the tissue is altered. Thus, the abnormal nutrient supplies to cells lead to progressive tissue degeneration [4].

Despite extensive efforts that focused on the development of computational models to predict* in vivo* cartilage behavior, and its application to clinical diagnosis and prognosis, substantial aspects nonetheless remain obscure.

Most of those studies have been done in 1D or 2D. However, nowadays, modern techniques of medical imaging, such as MRI, CT, MR, or PET, generate 3D images containing accurate information that cannot be captured by 1 or 2D models [19–21].

Besides,* in vivo* cartilage is influenced by real 3D boundary conditions [16, 22, 23]. Several reports demonstrate that cartilage properties (permeability, diffusivity, electric conductivity, and electrochemical potentials) are tightly interconnected with each other [24].

In this paper, we present a 3D mechano-electrochemical computational model of cartilage behavior, based on the triphasic theory of charged-hydrate soft tissues proposed by Lai et al. in 1991 [25] and lately extended by Sun et al. in 1999 [26].

The model has been used to the study of abnormal cartilage free swelling occurring in case of ALS patients. Similar to other cartilage disorders in immobilized patients, the degeneration process starts in the tissue surface. When the disease gets advanced, the degradation and subsequent dysfunction extend to deeper areas. The aim of this work is to focus on the response of this primarily affected zone to study its different behavior in healthy and pathological states [27–29].

The obtained results predict and quantify the impact of ALS immobilized cartilage properties into tissue deformation capacity which would enable monitoring the progress of cartilage tissue degeneration and evaluating the necessity of specific cartilage maintenance therapeutics during the different stages of the disease.

## 2. Material and Methods

The triphasic mechano-electrochemical theory [25] applied here considers a charged hydrated soft tissue as a mixture that consists of a porous permeable charged solid phase (ECM, collagen fibers, and PGs), a fluid phase (water), and an ion phase with two monovalent species (Na^+^ and Cl^−^). The main hypothesis of such triphasic theory assumes that the different phases exist simultaneously at each point in the space and are considered intrinsically incompressible.

This theory is mainly based on the balance equations (mass, momenta, and charge density) for the solid matrix, fluid (interstitial fluid), cations, and anions. Fluxes of fluid and ions depend on the gradients of their electrochemical potentials, which in turn, depend on fluid pressure and ion concentration, respectively.

### 2.1. Model Formulation

Four governing equations are required for solving the four basic unknowns in triphasic theory (**u** ^*s*^, the displacement of the solid matrix, *ε* ^*w*^, the modified chemical potential of water, and *ε* ^+^ and *ε* ^−^, the modified chemical potential for cations and anions, resp., [26]). These may be written as follows:

 Momentum balance equation of the mixture
  (1) $$\begin{array}{c} \nabla \cdot \sigma =0, \end{array}$$
 Mass balance equation of the mixture
  (2) $$\begin{array}{c} \nabla \cdot v^{s}+\nabla \cdot J^{w}=0, \end{array}$$
 Charge balance equation for each ion
  (3) ∂(Φwc+)∂t+∇·J++∇·(Φwc+vs)=0,
  (4) ∂(Φwc−)∂t+∇·J−+∇·(Φwc−vs)=0,

where **σ** is the mixture stress tensor and **v** ^*s*^ is the velocity of the solid matrix (small deformations has been considered here), specifically **v** ^*s*^ = ∂**u** ^*s*^/∂*t*, where ∂/∂*t* is the temporal derivative. *c* ^+^ and *c* ^−^ are cation and anion concentration, respectively, and Φ^*w*^ represents the cartilage porosity. **J** ^*w*^ is the water flux while **J** ^+^ and **J** ^−^ are the cation flux and anion flux, respectively. Importantly, we consider in our formulation the convective terms associated to ion-matrix coupling in the last part of the balance equations for the ions, (Φ^*w*^ *c* ^+^ **v** ^*s*^) and (Φ^*w*^ *c* ^−^ **v** ^*s*^). This is neglected in most cartilage models of this type in literature [30, 31], while they are essential for capturing the different biological coupled movement of the ions attached to collagen fibers through GAGs in healthy and degenerated tissues.

The constitutive equations for the state variables appearing in (1)–(4), *ε* ^*w*^, *ε* ^+^, and *ε* ^−^, in terms of the basic variables of the problem, may be also written as [15]

(5) σ=−PI+λsθI+2µsϵ,εw=PRT−Φ(c++c−)+BwRTθ,ε+=γ+c+exp⁡⁡(FcψRT),ε−=γ−c−exp⁡⁡(−FcψRT),

where *F*_*c*_ is the Faraday constant, *ψ* is the electrical potential, *B*_*w*_ is the fluid-solid coupling coefficient, Φ is the osmotic coefficient, *γ* ^+^ and *γ* ^−^ are the activity coefficient of anion and cation, respectively, and **I** is the identity tensor. *R* is the universal gas constant, and *T* is the absolute temperature.

Here, *P* is the fluid pressure, *θ* = div⁡**u** ^**s**^ is the solid matrix dilatation related to the infinitesimal strain tensor of the solid matrix, *λ*_*s*_ and *µ*_*s*_ are the Lamé constants of the solid matrix (a linear elastic isotropic material has been used for the sake of simplicity), **ϵ** = (1/2)(∇·**u** ^**s**^ + ∇^**T**^ · **u** ^**s**^) is the solid matrix deformation, and *c* ^+^ and *c* ^−^ can be expressed in terms of modified electrochemical potentials and fixed charged density as follows [26]:

(6) c+=cF+(c0F)2+(4ε+ε−/(γ+γ−))2,c−=−cF+(c0F)2+(4ε+ε−/(γ+γ−))2,

where *c*_0_^*F*^ is the initial FCD, the amount of charges which are attached to collagen fibers within the GAGs.

Regarding, **J** ^*w*^, **J** ^+^, and **J** ^−^, they can be expressed as combination of the chemical potential gradients as follows [26]:

(7) Jw=−RTΦwα(∇εw+c+ε+∇ε++c−ε−∇ε−),J+=−RTΦwc+α∇εw−[Φwc+D+ε++RTΦw(c+)2αε+]∇ε+−RTΦwc+c−αε+∇ε−,J−=−RTΦwc−α∇εw−[Φwc−D−ε−+RTΦw(c−)2αε−]∇ε−−RTΦwc+c−αε+∇ε+,

where *α* is the drag coefficient between the solid and the water phase, *D* ^+^ and *D* ^−^ the ion diffusivities, and Φ^*w*^ the porosity of the tissue.

The intrafibrillar water within collagen fibers, therefore, may be regarded as bound water and thus part of the solid phase. Due to the intrinsic incompressibility, for infinitesimal strains, Φ^*w*^ can be expressed as

(8) Φw=1−ϕ0s1+θ  ,

where *ϕ*_0_^*s*^ is the solid volume fraction at a physicochemical reference configuration (selected as the initial state). The second part of this equation, *ϕ*_0_^*s*^/(1 + *θ*), corresponds to the solid volume fraction of the tissue, *ϕ* ^*s*^, at any time along the deformation process. Note that, in (7), the combination of the porosity and the drag coefficient of the material give rise to the permeability of the tissue, *k*, as follows: *k* = Φ^*w*^/*α* [26].

Considering the electroneutrality condition between cations and anions, the following relation does exist [26]:

(9) c+=c−+cF,

where *c* ^*F*^ is the FCD. We assumed that the porous matrix is negatively charged, the fixed charges on the matrix remain unchanged, and *c* ^*F*^ must be conserved during the tissue deformation. According to experimental observations, increasing the volumetric deformation of the sample (e.g., tissue free swelling) is followed by a decrease in the fixed charge of the tissue as it involves water entrance and therefore ion concentration and fixed charges decrease in the matrix. Likewise, the opposite phenomenon also occurs when the volumetric deformation decreases (e.g., the tissue shrinks), under the following considerations [26]:

(10) cF=c0F1+(θ/Φ0w),

where *c*_0_^*F*^ and Φ_0_^*w*^ are the initial water content in the tissue at reference state.

#### 2.1.1. Differential Equation System

Substituting the flux equation (7) and the constitutive equation (5) into the governing equations (1)–(4), we find the following system of differential equations:

(11) ∇·(λsθI+2μsϵ)−∇(RTεw+RTΦck−Bwθ)=0,∇·vs−∇·RTα[Φw∇εw+Φwc+ε+∇ε++Φwc−ε−∇ε−]=0,∇·[−RTαΦwcF∇εw]−∇ ·[Φwc+D+ε++RTαΦw(c+)2ε+−RTαΦwc+c−ε+]∇ε+  +∇·[Φwc−D−ε−+RTαΦw(c−)2ε−−RTαΦwc+c−ε+]∇ε−   =0,∂(Φwck)∂t−∇·[−RTαΦwck∇εw]−∇ ·[Φwc+D+ε++RTαΦw(c+)2ε+−RTαΦwc+c−ε+]∇ε+−∇ ·[Φwc−D−ε−+RTαΦw(c−)2ε−−RTαΦwc+c−ε+]∇ε−+∇ ·(Φwckvs)=0,

where *c* ^*k*^ = *c* ^+^ + *c* ^−^.

#### 2.1.2. Solution Methods

A finite element formulation was used to obtain the solution of the governing system of (11). The primary unknowns of the model [**u**, *ε* ^*w*^, *ε* ^+^, *ε* ^−^] are interpolated from nodal values through shape functions [26]. The time derivatives are approximated with the Crank-Nicolson method [32] that yields an implicit approximation to the solution of the initial value problem *y*′ = *f*(*x*, *y*) with *y*(*x*_0_) = *y*_0_ at *x* for a given time step *h*. To obtain the fully coupled nonlinear system of equations describing the discretized model (superscript ∗ indicates the quantities in the bathing solution), we first establish the weak formulation of the governing equations:

(12) ∫∇δu·σ dV=∫Γδuσ∗n dΓ,∫∇δεw·vs dV+∫∇δεw·Jw dV=−∫ΓδεwJw∗n dΓ,∫∇δε+·J+ dV−∫Ω∇δε+·J− dV=∫Γδε+(J−∗−J+∗) n dΓ,∫δε−∂(Φwck)∂t dV+∫∇δε−·J+dV+∫∇δε−·J− dV  +∫∇δε−·(Φwckvs) dV=−∫Γδε−(J+∗+J−∗)n dΓ,

where **n** is the unit normal vector to the boundary and *δ*_**u**_, *δ*_*ε*^*w*^_, *δ*_*ε*^+^_, and *δ*_*ε*^−^_ are the so-called test functions. Trilinear 8-noded hexahedral elements with 2 × 2 × 2 Gaussian integration points were used (see Supplementary Material available online at http://dx.doi.org/10.1155/2014/179070).

### 2.2. Numerical Implementation of the Mechano-Electrochemical Model

**Figure 2:**
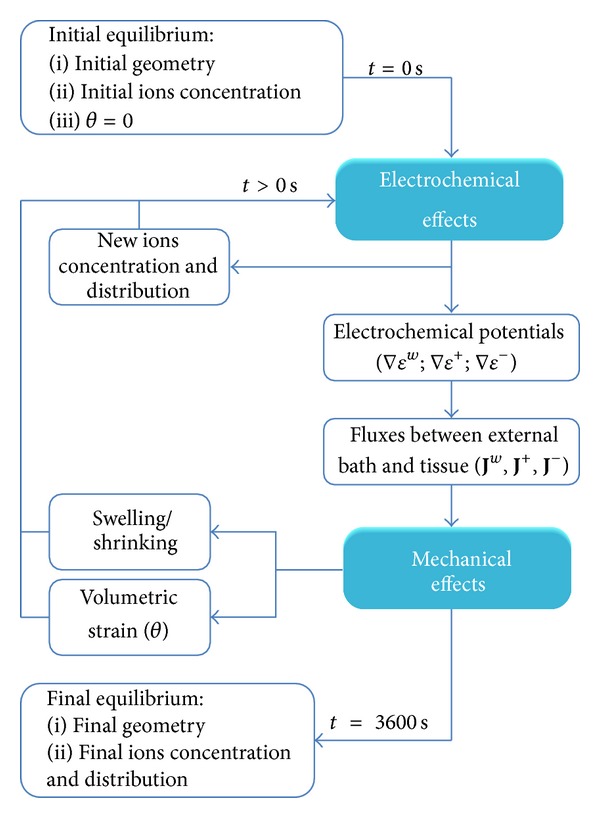
Schematic diagram of the computational process.

The model presented above has been implemented within a commercial FE software ABAQUS via a user defined subroutine. Initially, the cartilage sample is in equilibrium with the external bath (initial equilibrium). Therefore, the initial volumetric dilatation (*θ*) is equal to zero and initial values for ion concentrations correspond to those measured in the initial state of the external solution. After modifying the external solution, electrochemical potentials between the bath and the tissue are created (∇*ε* ^*w*^, ∇*ε* ^+^, and ∇*ε* ^−^), generating a new ion distribution within the cartilage. Due to this potential, fluxes of water and ions tend to enter and leave the tissue, respectively, to regain equilibrium and the tissue swells in absence of external loads. Specific values for displacement and ion concentrations are calculated in every node and updated via a Lagrangian formulation (in terms of the initial coordinates). The volumetric deformation at each finite element node is also computed. The full analysis presented in this work corresponds to a total time analysis (*t*_*f*_) of 3600 seconds coincident with usual experimental periods in cartilage free swelling assays [25]. The implementation scheme of the 3D mechano-electrochemical model is shown in Figure 2.

### 2.3. Experimental Example

Firstly, to validate the model, the experimental test described in [33] with healthy cartilage sample parameters is reproduce computationally. Then, the swelling of a cartilage sample of 0.5 mm high and 1.5 mm diameter confined in a chamber immersed in a NaCl solution was analyzed for healthy and degenerated cartilage tissue, including the properties associated to different stages of ALS patient joints suffering a progressive loss of motion.

#### 2.3.1. Boundary Conditions

**Figure 3:**
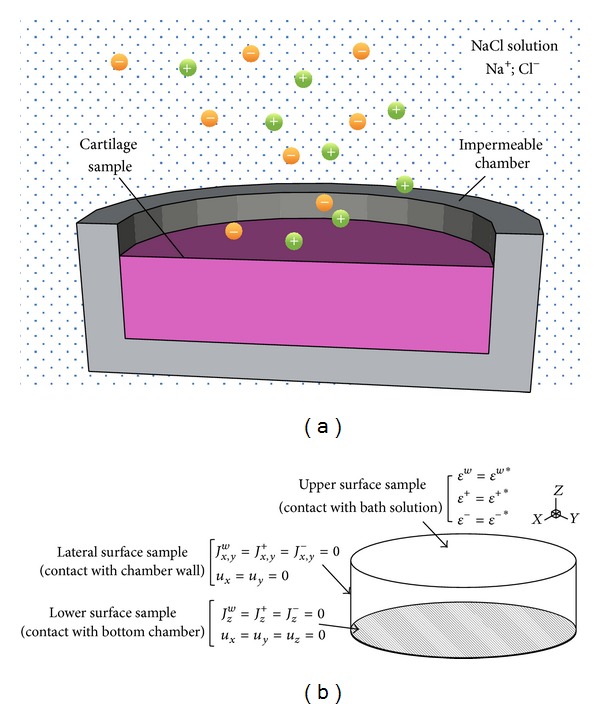
Schematic representation of the transient free swelling problem in Frijns et al. [33]. (a) A sample of cartilage of 0.5 mm thickness and 1.5 mm diameter is immersed in a NaCl solution with an initial concentration of 0.15 M. The sample is confined in an impermeable chamber. (b) Boundary conditions of the cartilage sample applied in our computational simulations.

The lateral sides of the sample as well as the supporting surface are impermeable and confine the lateral movement of the tissue (see Figure 2). Zero fluxes were considered for ions and water through these surfaces of the sample (Figure 3(b)). Specifically, for the lateral surfaces *J*_*x*,*y*_^*w*^ = *J*_*x*,*y*_^+^ = *J*_*x*,*y*_^−^ = 0 and for the lower surface, *J*_*z*_^*w*^ = *J*_*z*_^+^ = *J*_*z*_^−^ = 0. Furthermore, it is assumed that displacements in *x* and *y* directions remain null on lateral surfaces, *u*_*x*_ = *u*_*y*_ = 0. For the lower surface of the sample, we constrain all displacements, *u*_*x*_ = *u*_*y*_ = *u*_*z*_ = 0. Regarding the free upper surface of the sample, we consider the electrochemical potential of water and ions identical to those measured in the external bath, *ε* ^*w*^ = *ε* ^w*^; *ε* ^+^ = *ε* ^+*^; *ε* ^−^ = *ε* ^−*^. *ε* ^w*^, *ε* ^+*^, and *ε* ^−*^ in the external solution are kept constant in the whole process (no additional salt is added; no dilution occurs in the bathing solution).

#### 2.3.2. Initial Conditions

Initially it is considered that the sample is at equilibrium with the external bathing solution with a concentration *c*_0_* = 0.15 M of monovalent ions (Na^+^ and Cl^−^). At *t* = 0 s the concentration of the external solution is decreased to 0.125 M. This reduction causes the tissue to swell to a new equilibrium state (final equilibrium). The initial modified chemical potential for water *ε*_0_^w*^ and the electrochemical potential for cations and ions, *ε*_0_^+*^and *ε*_0_^−*^, inside the tissue are identical to their counterparts in the external solution, *ε*_0_^*w*^ = *ε*_0_^w*^; *ε*_0_^+^ = *ε*_0_^+*^; *ε*_0_^−^ = *ε*_0_^−*^. Choosing this free swollen state as the reference configuration (initial equilibrium), the initial displacement is measured from this reference state so, *u*_*x*_0__ = *u*_*y*_0__ = *u*_*z*_0__ = 0. The transient response of the solid displacement, ion concentration, fluid, and ion velocities to this imposed ionic change is solved here by using our 3D model and is compared with previous numerical results of this same example, obtained in 1D by Sun et al. (1999) [26].

### 2.4. Model Validation

In absence of 3D mechano-electrochemical computational models of cartilage in the literature, we first reproduced the results obtained by Sun et al. in one-dimension (1D) for healthy cartilage to validate our model. This 1D model includes an extensive study of the effects of cartilage parameter variations in tissue behavior and clearly states the differences and limitations found between their model and the experimental results.

Coincident with Sun's 1D model, the mesh of our model presents a total number of elements equal to 50 in depth. To accurately reproduce the 1D case of study, we consider a diameter four orders of magnitude smaller than the height of the sample (*∅*_sam_ ≪ *h*_sam_), with only one element per row. The selected average mesh size was of 0.01 mm. Finally, the resulting total number of elements is 50. Similar to Sun et al. (1999) [26], the displacements in *x* and *y* axis are constrained into the whole sample (*u*_*x*_ = *u*_*y*_ = 0) and therefore, analyzing only *z* displacement (*u*_*z*_) effects. All parameters used in this example are collected in Table 1. However, Sun's experimental results and its measurements variability are not indicated in their work, they state that simulated surface displacement in free swelling test is consistent with their experimental observations [26]. Their computed and experimental results have been used to validate the presented mechano-electrochemical model.

### 2.5. 3D Free Swelling Study of Healthy and Immobilized ALS Patients Cartilage

The model was applied to analyze the effect of the variation of different properties governing cartilage behavior in patients with lack of mobility as in ALS patients. In this model application, the experimental conditions detailed in Frijns et al. (1997) [33] again motivated the geometry of the confined sample (*∅*_sam_ = 1.5 mm), with simulations that particularly considered the interplay between all properties interacting in a 3D environment. Results corresponding to healthy cartilage were compared to those yielded by Sun's 1D model and biological studies for healthy cartilage behavior. Furthermore, we obtained accurate quantification of water and ion fluxes of these samples. Then, a comparative study between healthy and degraded cartilage free swelling was performed.

An interesting phenomenon of a delay in cartilage swelling, associated with a small, effectively zero, amount of shrinking was observed in our simulation during the initial phases when values of FCD into the tissue or Φ are coincident with those measured in unhealthy cartilage tissue [24]. This effect has not previously observed with 1D or 2D models applying a wide variety of FCD for progressive immobilized knees [26], which ranged from degenerated aged knees (e.g., *c*_0_^*F*^ = 0.11 mEq/mL) to tissues with other degenerative diseases as osteoarthritis (e.g., *c*_0_^*F*^ = 0.06 mEq/mL).

## 3. Results and Discussion

The present model was used to simulate the behavior of healthy cartilage in free swelling conditions. Then, additional simulation were performed varying physiological tissue properties (porosity, osmotic coefficient, and fixed anions into the tissue) to simulate progressive immobilization process that take place in disabled ALS patients. The resulting abnormal cartilage free swelling was compared with that obtained with healthy tissue.

Initially, we analyzed the *z*-displacements of the upper surface of the cartilage sample as well as cation distribution within the sample after changing the concentration of the external solution from 0.15 M to 0.125 M. As previously mentioned, for validation the 3D model was simplified reducing the sample diameter to mimic 1D model conditions. Results obtained were compared to those reported by Sun et al. (1999) [26] with their 1D model. Subsequently, the same situation was reanalyzed considering the 3D environment and applying physiological versus pathological cartilage properties, real sample dimensions, and three-dimensional confined conditions (see Table 2).

### 3.1. 3D Simplified Model

**Figure 4:**
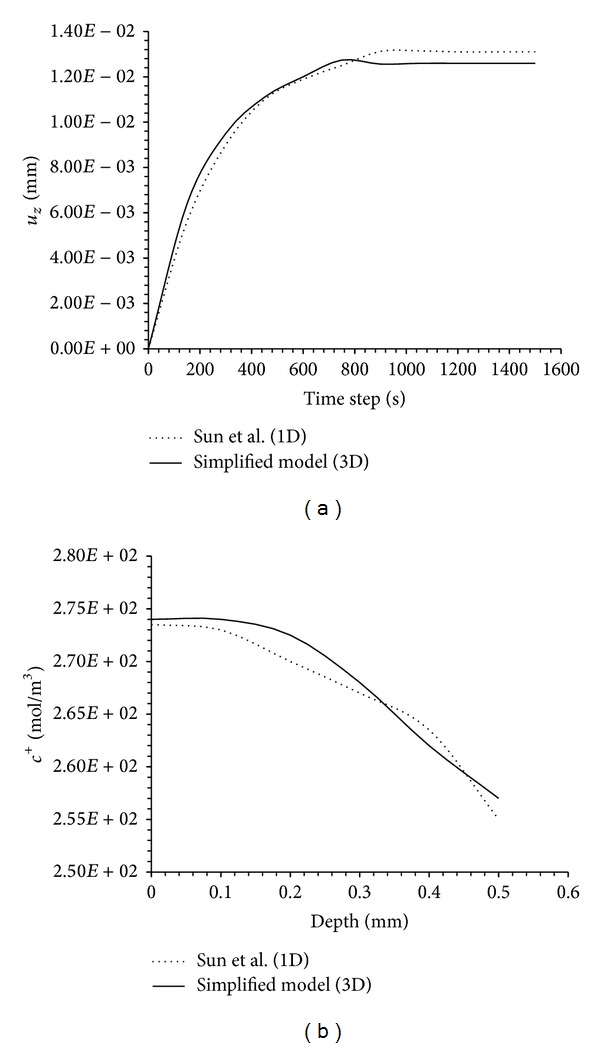
(a) Comparison of the upper surface displacement in the free swelling test from 1D model of Sun et al. (dotted line) and the present 3D simplified model (continuous line). (b) Comparison of cation concentration distribution in the free swelling test from 1D model of Sun [26] and the present 3D simplified model after 100 seconds of swelling.

Under the assumption of frictionless, chamber-impermeable walls and bottom surface, the displacement of the upper surface is only affected by entrance of water and exit of ion fluxes. The simplified 3D model shows that, after dilution and the associated massive entrance of water into the sample, the cartilage begins to swell reaching its maximal deformation at 900 seconds and 0.013 mm height. After this point, sample dimensions remain constant and enter a plateau stage (Figure 4(a)). The present simplified 3D model accurately reproduced the deformation evolution obtained by Sun who found a maximal deformation of 0.0135 mm height at 1000 seconds, with the subsequent steady state with that dimension.

In these conditions the gradient of cations was also monitored after 100 seconds of this free swelling simulation and again compared well with the results reported by Sun 1D model. At that time, the 1D model clearly showed a progressive decrease of cation concentration towards the upper surface of the sample (*x* = 0.5 mm) ranging from 273 mol/m^3^ at *x* = 0 mm height (significantly lower compared to the initial 280 mol/m^3^ imposed by the external solution at time 0 s) to 255 mol/m^3^ at *x* = 0.5 mm height (Figure 4(b)).

Our results closely resemble those obtained by Sun with an initial concentration of 274 mol/m^3^ at *x* = 0 mm height and 257 mol/m^3^ at *x* = 0.5 mm height (Figure 4(b)).

### 3.2. 3D Free Swelling Study of Healthy and Immobilized ALS Patients Cartilage

In this study the experiment described by Sun et al. was again reproduced considering a sample diameter of 1.5 mm and the 3D confined environment reported experimentally by Frijns et al. (1997) [33] together with the physiological properties of cartilage later presented in Sun et al. in 2004 [15] (Table 2).

Under these conditions, fluxes of water, cations, and anions during the whole process were computed, monitored, and analyzed. In addition, displacements of the free healthy cartilage surface were again simulated, as well as the effects of the variation in the main cartilage properties (fixed charge density, porosity of the tissue, and ion diffusivities) according to the grade of mobility and total immobilization time of ALS patients [34].

#### 3.2.1. Fluxes of Water and Ions in Healthy Cartilage

Concurrent water and ions fluxes, together with morphological changes of the tissue were simulated during 3600 seconds. The corresponding simulation results were divided into five phases corresponding to the main events that take place experimentally [35]. A detailed description of the status of each component during these phases is as follows.

**Figure 5:**
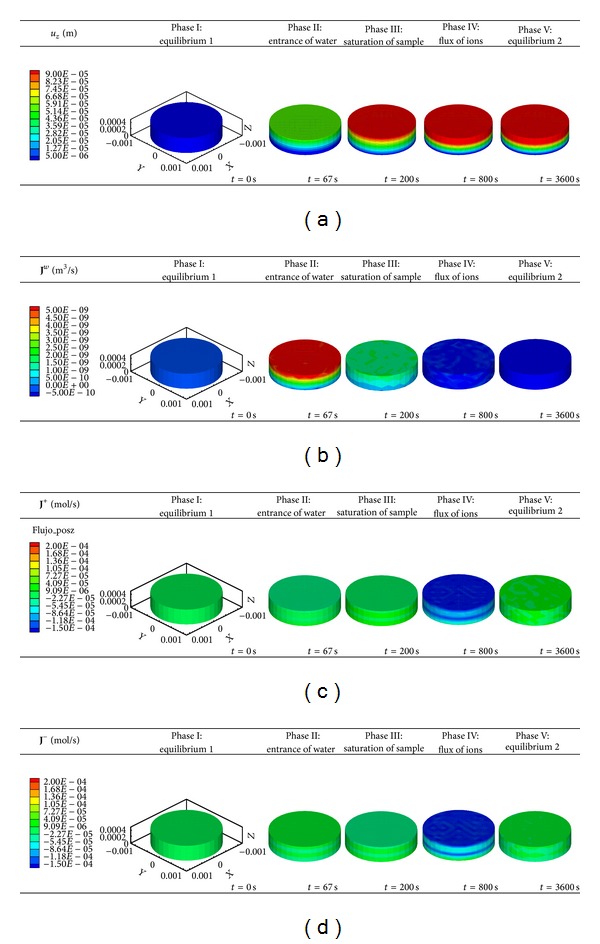
(a) *z*-displacement (*u*_*z*_), (b) water (**J** ^*w*^), (c) cation (**J** ^+^), and (d) anion (**J** ^−^) fluxes obtained with the presented 3D computational model during five phases of swelling (3600 seconds total time simulation) in healthy cartilage samples. Note that negative fluxes for each species studied correspond to the emergence of that component from the sample to the external solution. Conversely, positive fluxes refer to the entrance of the different components (water or ions) into the sample (tissue gain of material).

*Phase I (initial equilibrium)*. In this stage, the external solution is diluted to 0.125 M of NaCl which generates an imbalance between the inner and outer medium of the sample. At time 0 the sample remains equilibrated with the 0.15 M of NaCl initial solution. 3D computational results did not reveal any deformation (Figure 5(a), Phase I) as water (Figure 5(b), Phase I) and ion fluxes (Figures 5(c) and 5(d), Phase I) are minimal as well.

*Phase II*. This phase represents an abrupt deformation of the cartilage (Figure 5(a), Phase II) as a consequence of the massive entrance of water at 5.0 · 10^−9^ m^3^/s (Figure 5(b), Phase II) to counteract the gradient induced in the previous step. At this stage, outgoing ion fluxes would be expected; however, these are clearly overwhelmed by the intake water flux (Figures 5(c) and 5(d), Phase II), which leads to subsequent substrate swelling during the next phase.

*Phase III*. During this phase, high displacement of the upper surface of the sample (Figure 5(a), Phase III) is observed at 200 seconds as already mentioned. This is due to the previous and continuous water flux uptake during this stage (Figure 5(b), Phase III), when the maximum water content within the sample is reached. Consistently, ion fluxes for both cation (Figure 5(c), Phase III) and ions (Figure 5(d), Phase III) remain at basal level.

*Phase IV*. After 800 seconds of simulation, the substrate deformation is maintained (Figure 5(a), Phase IV); water flux is drastically reduced (Figure 5(b), Phase IV) while internal and external solutions regain the equilibrium state, which enables the visualization of the previously impeded ion outflow (Figures 5(c) and 5(d), Phase IV).

*Phase V (final equilibrium)*. Finally, this phase represents the complete restoration of electrochemical equilibrium between the external and internal solution. The cartilage sample does not show any further deformation (Figure 5(a), Phase V), in agreement with no external load applied on the sample and the lack of a Na, Cl ion gradient. Consistently, water (Figure 5(b), Phase V) and ion fluxes (Figures 5(c) and 5(d), Phase V) were not shown at this stage.

#### 3.2.2. Effects of Initial Fixed Charge Density (FCD)

To fully understand the effect of FCD on swelling, the following experiment was designed. Initial FCD measured experimentally in a young and aged healthy knees cartilage (*c*_0_^*F*^ = 0.2 mEq/mL and *c*_0_^*F*^ = 0.135 mEq/mL, resp.) [24] and three different values of *c*_0_^*F*^ in patient with osteoarthritis and cartilage degenerative processes where the patient must remain partially (early stage of ELA) or totally immobilized (advanced stage of ELA) [34, 36] were introduced into the present 3D computational model to compare the corresponding results.

**Figure 6:**
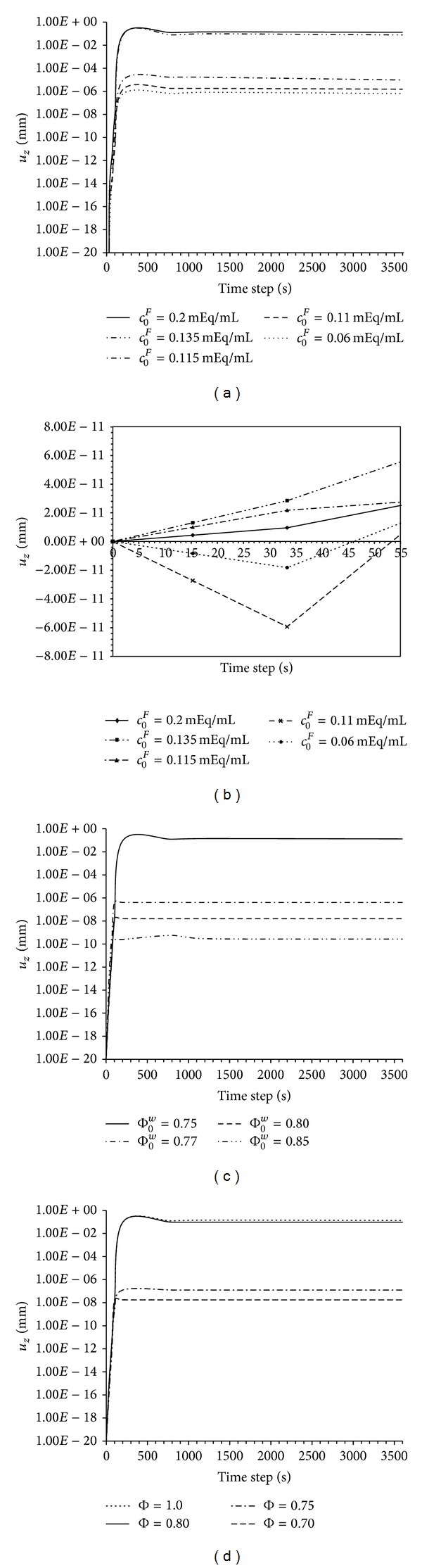
(a) Surface displacement obtained with the 3D computational model as a function of tissue FCD. The rest of the model parameters are included in Table 2. (b) Details of the initial swelling delay phenomenon of cartilage associated with a nominal shrinking within the first 40 seconds of computational simulation considering the parameters collected in Table 2, while the specific concentration of FCD shown in the same figure. (c) Surface displacement obtained with the 3D computational model as a function of tissue initial water content (Φ_0_^*w*^). (d) Surface displacement obtained with the 3D computational model as a function of the osmotic coefficient.

The results showed that physiological FCD values displayed a maximal displacement of 0.143 mm for young healthy knee and 0.0984 mm for aged healthy knee within 200 seconds of simulation, subsequently these values remained constant for 3600 seconds. The rest of FCD selected values (representative of pathologic cartilage conditions) offered lower *z*-displacement, ranging from 1.679 · 10^−5^ mm to 8.218 · 10^−7^ mm (i.e., almost null swelling), after 900 seconds of simulation time (Figure 6(a)). In every case, after tissue researches its maximum *z*-displacement value, this state was maintained until the end of the simulation (3600 seconds).

It is of interest to note that the present model shows a delay in subsequent swelling associated with a tiny, effectively zero initial shrinking which appears when introducing the lower values of FCD content. This phenomenon is illustrated in the three studies (variation of FCD, porosity of the tissue, and osmotic coefficient) (see Figure 6(b)) but was not observed with higher FCD values.

#### 3.2.3. Effects of Initial Water Content

The influence of initial porosity in the *z*-displacement suffered by the tissue was also simulated. This porosity directly correlates with the initial amount of water in the cartilage sample since a fully saturated sample is assumed. For a normal human knee cartilage this ranges from 75 to 78% [37].

Results demonstrated that, similarly to FCD influence, a physiological initial porosity of the tissue (Φ_0_^*w*^ = 75% of water content) yielded the highest displacement at the upper surface of the sample, which exhibited a sharp increment within the first 200 seconds of simulation and peaked at 0.143 mm. Again the volume of the sample remained constant after this time (Figure 6(c)).

High initial porosities in the tissue (which imply higher water content) leaded to proportional lower maximal displacements (Figure 6(c)). Interestingly, several harmful conditions to the maintenance of cartilage such as the loss of motion that ELA patients suffer involve the reduction in porosity [34]. High water content in these tissues may limit the capture of additional water molecules and therefore downregulate in going fluxes as discussed below.

#### 3.2.4. Effects of Osmotic Coefficient

If we now modify the osmotic coefficient of solid matrix and fix the rest of the parameters (Table 2), it is possible to quantify the influence of this variable in the swelling of the cartilage sample. Values below the physiological threshold, Φ = 0.8, fail to exhibit the normal-range deformation of the substrate, 0.143–0.0954 mm at 200 seconds of simulation (Figure 6(d)). Biologically, this coefficient affects the velocity of water flow through the porous medium. Thus, a reduction of this coefficient, often under critical conditions such as joint large immobilization periods of time [38, 39] entails a decrease of fluid flow into the cartilage porous matrix and therefore implies smaller deformations.

## 4. Conclusion

In this work a 3D mechano-electrochemical cartilage behavior model is presented. The model has been applied to study the cartilage free swelling that takes place in case of ALS patients by varying the main tissue properties (porosity, osmotic coefficient, and fixed anions).

Specifically, in this study we analyze and quantify the effect of FCD, porosity of the ECM, ionic diffusivity, electrochemical potentials, and mechanical ECM properties in the behavior of the cartilage tissue, ranging from healthy cartilage properties to those associated with joints from ALS patients at different stages.

Several simplifications have been made to develop this model. Firstly, we have neglected the PGs effects due to FCD repulsion and the anisotropy in the mechanical behavior of cartilage [40]. Including these aspects would validate the proposed model for wider purposes; however, it would also increase the number of model parameters and the associated validation. Secondly, the specific values for the different variables included in the model (Table 2) were taken from experimental reports in literature which generate a remarkably wide physiological range for each variable considering the very different measurement tools used in each study and the technical limitations of these quantifications. Moreover, parameters such as age, sex, or weight of the donor may severely influence these variables [24]. Under these conditions, the model has been validated including different cases within the physiological range for each variable; those parameters that had been reported to drastically influence the value of a specific variable were analyzed separately (i.e., healthy versus Immobilized *c*_0_^*F*^ in cartilage knees) (Figure 6(a)).

In absence of literature about previous models and accurate free swelling measurements in a 3D geometry, we validated the presented model by comparing the results with the 1D simulation of Sun et al. (1999) [26]. As expected, similar swelling displacements were obtained in both cases, although, in the full 3D model, swelling occurs faster and the new equilibrium (final equilibrium) after changing the external solution concentration is reached earlier. These differences may arise from the extensive simplifications made by Sun et al. such as neglecting the effect of osmotic coefficient and ions activities to get convergence. In this sense, even including the values reported by Sun et al. our results closely parallel those obtained by Frijns et al. (1997) [33], who reported an earlier and faster swelling. By including the same parameter values reported in Frijns' experimental work within the 3D model, the maximum surface displacement slightly increased together with the swelling velocity, as reported by these authors.

In the full 3D free swelling study applied to health and ALS-degraded cartilage due to loss motion, computational results showed how FCD ranging within the physiological value (0.2 mEq/mL and 0.135 mEq/mL) results in sharp swelling. However, reducing FCD content, according to ALS patients with progressive loss motion, it reduces deformation rates. Small variations in the FCD content would lead to high deviations of the swelling rate. FCDs below the physiological threshold have been described in arthritical, degenerated or unhealthy cartilage [41]; therefore, tissue deformation under these conditions would be impaired in agreement with our computational observations.

Due to its 3D consideration, the modelling framework is sensitive to radial boundary conditions as occurs experimentally. Specifically, in the 3D model, the initial swelling delay is induced not only for values of FCD lower than 0.05 mEq/mL, as Sun et al. mentioned, but also for values of 0.11 mEq/mL. The increased sensitivity of our model is possibly due to the inclusion of 3D boundary conditions. In any case, it is remarkable that nominal shrinking and an associated delay in significant net response was induced when including small FCDs. We speculate that, biologically, in these conditions higher amounts of mobile ions remain uncoupled, increasing the fluxes of cations and anions. Considering that, immediately after dilution of the external solution to 0.125 M high ion fluxes arise from the more concentrated internal solution during the initial stages, before water entrance overwhelms this flux, inducing a delay in the net cartilage response and a tiny, effectively negligible, shrinking. The effect of initial porosity (Φ_0_^*w*^) and osmotic coefficient (Φ) on the swelling of the sample is also enhanced by the 3D conditions. Again upper surface cartilage decreases, inducing a delay in the onset of swelling, when the model adopts subphysiological values of the porosity or osmotic coefficient, Φ = 0.75 and Φ = 0.8, respectively. This is explained by considering that all properties are interconnected within the coupled 3D computational model; hence, the disturbance of one of them highly influences the rest, increasing the stiffness of the tissue and impeding the normal damping of the charges [24].

Since swelling is a key process in cartilage tissue [18, 42, 43], this event was thoroughly analyzed within the three-dimensional study of water and ion fluxes. After changing the concentration of the external solution, initial equilibrium was broken and an incoming flux of water into the sample arises to re-equilibrate both solutions. The abrupt water entrance results in sharp swelling of the sample for physiological parameters. During this stage, water flux hides the comparatively minor outgoing ion fluxes. However, during the later stage this last flow becomes apparent, as water current decreases.

In summary, the presented 3D model is able to decipher the effects on cartilage deformation impairment for nonphysiological values of the osmotic coefficient, porosity, and fixed charges density, all of which are measurable parameters. The obtained results predict and quantify the impact of ALS immobilized cartilage properties into tissue deformation capacity. All these features, together with the display of results into clinically interpretable 3D images present this model as a helpful tool to predict, prevent, and monitor the progress of cartilage tissue degeneration in ALS patients and abrogates for the inclusion of cartilage protective therapy into the management of the disease to ameliorate the motion loss.

## Supplementary Material

The finite element weak formulation and spatial discretization of the model governing equations.

## Figures and Tables

**Table 1:** 3D simplified model for healthy cartilage parameters.

Description	Symbol	Value	Refs
Initial thickness of the sample	*h*_sam_	0.5 mm	[26]
Initial diameter of the sample	*∅*_sam_	0.0005 mm	—
Number of mesh elements	NELEM	50	[26]
Number of mesh nodes	NNODES	204	—
Young's modulus	*E*	3.85 · 10^5^ Pa	[15]
Poisson coefficient	*ν*	0.28	[15]
Drag coefficient between the solid and the water phase	*α*	7 · 10^14^ N·s·m^−4^	[26]
Diffusivity of the cations	*D* ^+^	5 · 10^−10^ m·s^−1^	[26]
Diffusivity of the anions	*D* ^−^	8 · 10^−10^ m·s^−1^	[26]
Initial FCD	*c*_0_^*F*^	0.2 mEq·mL^−1^	[26]
Activity coefficient of cations	*γ* ^+^	1.0	[26]
Activity coefficient of anions	*γ* ^−^	1.0	[26]
Gas constant	*R*	8.314 J·mol^−1^·K^−1^	[26]
Absolute temperature	*T*	298 K	[26]
Osmotic coefficient	Φ	1.0	[15]
Initial amount of water in the tissue	Φ_0_^*w*^	0.75	[26]

**Table 2:** 3D free swelling model for healthy cartilage parameters.

Description	Symbol	Value	Refs
Initial thickness of the sample	*h*_sam_	0.5 mm	[26]
Initial diameter of the sample	*∅*_sam_	1.5 mm	[26]
Number of mesh elements	NELEM	760	[26]
Number of mesh nodes	NNODES	1026	—
Young's modulus	*E*	3.85 · 10^5^ Pa	[15]
Poisson coefficient	*ν*	0.28	[15]
Drag coefficient between the solid and the water phase	*α*	7 · 10^14^ N·s·m^−4^	[26]
Diffusivity of the cations	*D* ^+^	5 · 10^−10^ m·s^−1^	[26]
Diffusivity of the anions	*D* ^−^	8 · 10^−10^ m·s^−1^	[26]
Initial FCD	*c*_0_^*F*^	0.2 mEq·mL^−1^	[26]
Activity coefficient of cations	*γ* ^+^	0.86	[6]
Activity coefficient of anions	*γ* ^−^	0.85	[6]
Gas constant	*R*	8.314 J·mol^−1^·K^−1^	[26]
Absolute temperature	*T*	298 K	[26]
Osmotic coefficient	Φ	0.8	[6]
Initial amount of water in the tissue	Φ_0_^*w*^	0.75	[26]
