# Supplementary material for: Cartilage Dysfunction in ALS Patients as Side Effect of Motion Loss: 3D Mechano-Electrochemical Computational Model
Source: Biomed Res Int. 2014 Jun 3;2014:179070. doi: 10.1155/2014/179070 (PMC4065674; doi:10.1155/2014/179070)
Supplement: Supplementary file 1 — The finite element weak formulation and spatial discretization of the model governing equations. [file 179070.f1.pdf]

Weak formulation of mechano-electro-chemical cartilage model

Discretization and non-linear equations system

For the temporal discretization of the governing equations, we consider the partition $\cup_{n=1}^{n_{step}-1}[t_n, t_{n+1}]$ of the time interval of interest T, and focus on the typical time subinterval $[t_n, t_{n+1}]$ with $\Delta t = t_{n+1} - t_n \geq 0$ denoting the corresponding increment of time. It is assumed that the primary unknowns and all derivable quantities are known at time t_n. The generalized trapezoidal method is applied (Crank and Nicolson 1947), with $\alpha \epsilon (0,1]$, such that $t_{n+\alpha} = t_n + \alpha \Delta t$. In this method the following scheme is used for the temporal discretization of primary variables (here only ε^w is shown, while ε^+ and ε^- and $\mathbf{u}$ have the same discretization):

$$\varepsilon_{n+1}^{w} = \varepsilon_{n}^{w} + \Delta t\, \dot{\varepsilon}_{n+\alpha}^{w}\,, \tag{A.1}$$

$$\dot{\varepsilon}_{n+\alpha}^{w} = \dot{\varepsilon}_{n}^{w}(1-\alpha) + \dot{\varepsilon}_{n+1}^{w}\alpha\,, \tag{A.2}$$

where ε_{n+1}^{w}, $\dot{\varepsilon}_{n+\alpha}^{w}$ and $\dot{\varepsilon}_{n+1}^{w}$ are approximations of $\varepsilon^{w}(t_{n+1})$, $(\frac{\partial \varepsilon^{w}}{\partial t})(t_{n+\alpha})$ and $(\frac{\partial \varepsilon^{w}}{\partial t})(t_{n+1})$, respectively. From a practical standpoint $\tilde{\varepsilon}_{n+1}^{w}$ is introduced as a predictor value of ε_{n+1}^{w}, which only depends on magnitudes at time t_n:

$$\tilde{\varepsilon}_{n+1}^{w} = \varepsilon_{n}^{w} + (1-\alpha)\Delta t \cdot \dot{\varepsilon}_{n}^{w}\,, \tag{A.3}$$

$\dot{\varepsilon}_{n+1}^{w}$ can be computed by

$$\dot{\varepsilon}_{n+1}^{w} = \frac{\varepsilon^{w}{}_{n+1} - \tilde{\varepsilon}_{n+1}^{w}}{\alpha \Delta t}\,, \tag{A.4}$$

Substitution of equations (A.3) and (A.4) in the weak form of the problem yields a semi-discrete set of equations that are discretized in time.

Spatial discretization of the problem

The semi-discrete system is discretized in space using the finite element method. The domain Ω is discretized into n_{el} elements Ω^e, with $\Omega = \cup_{e=1}^{n_{\mathrm{el}}} \Omega^e$. The primary unknown fields are interpolated within a generic element Ω^{e} in terms of the nodal values through shape functions, that is,

$$\varepsilon^{wh}|_{\Omega^e} = \mathbf{N}_{\varepsilon^w} \boldsymbol{\varepsilon}^{w\mathrm{e}} \,,$$

$$\varepsilon^{+h}|_{\Omega^e} = \mathbf{N}_{\varepsilon^+} \boldsymbol{\varepsilon}^{+\mathrm{e}} \,, \qquad \text{(A.5)}$$

$$\varepsilon^{-h}|_{\Omega^e} = \mathbf{N}_{\varepsilon^-} \boldsymbol{\varepsilon}^{-\mathrm{e}} \,,$$

$$\mathbf{u}^h|_{\Omega^e} = \mathbf{N}_{\mathbf{u}} \mathbf{u}^e,$$

where $\boldsymbol{\varepsilon}^{w\mathrm{e}}$, $\boldsymbol{\varepsilon}^{+\mathrm{e}}$, $\boldsymbol{\varepsilon}^{-\mathrm{e}}$ and $\mathbf{u}^{\mathrm{e}}$ are column vectors of nodal values of the primary unknowns at element e and $\mathbf{N}_{\varepsilon^{\mathrm{w}}}$, $\mathbf{N}_{\varepsilon^+}$, $\mathbf{N}_{\varepsilon^-}$ and $\mathbf{N}_{\mathbf{u}}$ are matrices of element shape functions, that is,

$$\mathbf{N}_{\varepsilon^{\mathrm{w}}} = [N_{\varepsilon^w}^1, \dots, N_{\varepsilon^w}^{n_{en}}] \,,$$

$$\mathbf{N}_{\varepsilon^+} = [N_{\varepsilon^+}^1, \dots, N_{\varepsilon^+}^{n_{en}}] \,, \qquad \text{(A.6)}$$

$$\mathbf{N}_{\varepsilon^-} = [N_{\varepsilon^-}^1, \dots, N_{\varepsilon^-}^{n_{en}}] \,,$$

$$\mathbf{N}_{\mathbf{u}} = \begin{bmatrix} N_u^1 & 0 & 0 \\ 0 & N_u^1 & 0 \\ 0 & 0 & N_u^1 \end{bmatrix} \cdots \begin{bmatrix} N_u^{n_{en}} & 0 & 0 \\ 0 & N_u^{n_{en}} & 0 \\ 0 & 0 & N_u^{n_{en}} \end{bmatrix},$$

where $\mathbf{N}^{\mathrm{i}}$ is the shape function associated with element node i and n_{en} is the number of element nodes. Following a Bubnov-Galerkin scheme, the same shape functions are also applied to interpolate the test functions:

$$\delta\varepsilon^{wh}|_{\Omega^e} = \mathbf{N}_{\varepsilon^w}\boldsymbol{\delta\varepsilon}^{\boldsymbol{we}} ,$$

$$\delta\varepsilon^{+\mathrm{h}}|_{\Omega^e} = \mathbf{N}_{\varepsilon^+}\boldsymbol{\delta\varepsilon}^{+\boldsymbol{e}} , \tag{A.7}$$

$$\delta\varepsilon^{-h}|_{\Omega^e} = \mathbf{N}_{\varepsilon^-}\boldsymbol{\delta\varepsilon}^{-\boldsymbol{e}},$$

$$\delta\mathbf{u}^h|_{\Omega^e} = \mathbf{N}_{\mathbf{u}}\delta\mathbf{u}^e .$$

Likewise, the discretization of the related gradients of the test functions and the primary unknowns take the following element wise format:

$$\nabla\varepsilon^{wh}|_{\Omega^e} = \nabla\cdot\mathbf{N}_{\varepsilon^w}\boldsymbol{\varepsilon}^{w\mathrm{e}} \xrightarrow{yields} \nabla\delta\varepsilon_h|_{\Omega^e} = \nabla\cdot\mathbf{N}_{\varepsilon^w}\delta\boldsymbol{\varepsilon}^{w\mathrm{e}} , \tag{A.8}$$

$$\nabla\varepsilon^{+h}|_{\Omega^e} = \nabla\cdot\mathbf{N}_{\varepsilon^+}\boldsymbol{\varepsilon}^{+\mathrm{e}} \xrightarrow{yields} \nabla\delta\varepsilon^{+}{}_{h}|_{\Omega^e} = \nabla\cdot\mathbf{N}_{\varepsilon^+}\delta\boldsymbol{\varepsilon}^{+\mathrm{e}},$$

$$\nabla\varepsilon^{-h}|_{\Omega^e} = \nabla\cdot\mathbf{N}_{\varepsilon^-}\boldsymbol{\varepsilon}^{-\mathrm{e}} \xrightarrow{yields} \nabla\delta\varepsilon^{-}{}_{h}|_{\Omega^e} = \nabla\cdot\mathbf{N}_{\varepsilon^-}\delta\boldsymbol{\varepsilon}^{-\mathrm{e}} .$$

The strains are interpolated in the following form:

$$\epsilon^h|_{\Omega^e} = \mathbf{B}_{\mathbf{u}}\mathbf{u}^e , \tag{A.9}$$

where $\mathbf{B}_{\mathbf{u}}$ is a matrix of derivatives of shape functions:

$$\mathbf{B}_{\mathbf{u}} = \mathbf{H}\mathbf{N}_{\mathbf{u}} ,$$

(A.10)

$$\mathbf{H}=\begin{bmatrix}\frac{\partial}{\partial \mathrm{x}} & 0 & 0\\ 0 & \frac{\partial}{\partial \mathrm{y}} & 0\\ 0 & 0 & \frac{\partial}{\partial \mathrm{z}}\\ \frac{1}{2}\frac{\partial}{\partial \mathrm{y}} & \frac{1}{2}\frac{\partial}{\partial \mathrm{x}} & 0\\ \frac{1}{2}\frac{\partial}{\partial \mathrm{y}} & 0 & \frac{1}{2}\frac{\partial}{\partial \mathrm{y}}\\ 0 & \frac{1}{2}\frac{\partial}{\partial \mathrm{z}} & \frac{1}{2}\frac{\partial}{\partial \mathrm{y}}\end{bmatrix}. \tag{A.11}$$

Substituting the equations (A.5), (A.7) and (A.10) into the semi-discrete system and choosing appropriately arbitrary coefficients $\delta\boldsymbol{\varepsilon}^{we}$, $\delta\boldsymbol{\varepsilon}^{+e}$, $\delta\boldsymbol{\varepsilon}^{-e}$ and $\delta\mathbf{u}^{e}$ of the test functions, one can finally arrive at a set of non-linear algebraic equations which is sufficient to determine the nodal values of the primary unknowns that can be written in the form:

$$\mathbf{F}^{\mathrm{int}}=\left(\mathbb{Z}_{n+1},\frac{\mathbb{Z}_{n+1}-\tilde{\mathbb{Z}}_{n+1}}{\alpha\Delta t}\right)=\mathbf{F}^{\mathrm{ext}}(\mathbb{Z}_{n+1})\,, \tag{A.12}$$

where $\mathbb{Z}_{n+1}$ and $\tilde{\mathbb{Z}}_{n+1}$ are the global column vector of nodal values of the primary unknown fields at time $t_{\mathrm{n+1}}$ and the corresponding predictor value, respectively. This vector can be obtained as follow:

$$\mathbb{Z}_{n+1}=\mathcal{R}_{e=1}^{n_{\mathrm{en}}}\mathbf{d}_{n+1}^{e}\,, \tag{A.13}$$

$$\tilde{\mathbb{Z}}_{n+1}=\mathcal{R}_{e=1}^{n_{\mathrm{en}}}\tilde{\mathbf{d}}_{n+1}^{e}\,, \tag{A.14}$$

where $\mathcal{R}$ denotes the standard finite element assembly operator and $\mathbf{d}_{n+1}^{e}$ and $\tilde{\mathbf{d}}_{n+1}^{e}$ can be defined by

$$\mathbf{d}_{n+1}^{e}=[\boldsymbol{\varepsilon}_{n+1}^{we},\boldsymbol{\varepsilon}_{n+1}^{+e},\boldsymbol{\varepsilon}_{n+1}^{-e},\mathbf{u}_{n+1}^{e}]^{\mathrm{T}}, \tag{A.15}$$

$$\tilde{\mathbf{d}}_{n+1}^{e}=[\tilde{\boldsymbol{\varepsilon}}_{n+1}^{we},\tilde{\boldsymbol{\varepsilon}}_{n+1}^{+e},\tilde{\boldsymbol{\varepsilon}}_{n+1}^{-e},\tilde{\mathbf{u}}_{n+1}^{e}]^{\mathrm{T}}. \tag{A.16}$$

The internal and external global force vector represented by F^{int} and F^{ext} also come from the assembly of element contributions:

$$F_{n+1}^{int} = \mathcal{R}_{e=1}^{n_{en}} \mathbf{f}_{n+1}^{int,e} , \tag{A.17}$$

$$F_{n+1}^{ext} = \mathcal{R}_{e=1}^{n_{en}} \mathbf{f}_{n+1}^{ext,e} , \tag{A.18}$$

where

$$\mathbf{f}_{n+1}^{int,e} = [\mathbf{f}_{\varepsilon^w,n+1}^{int,e}; \mathbf{f}_{\varepsilon^+,n+1}^{int,e}; \mathbf{f}_{\varepsilon^-,n+1}^{int,e}; \mathbf{f}_{\mathbf{u},n+1}^{int,e}]^T , \tag{A.19}$$

$$\mathbf{f}_{n+1}^{ext,e} = [\mathbf{f}_{\varepsilon^w,n+1}^{ext,e}; \mathbf{f}_{\varepsilon^+,n+1}^{ext,e}; \mathbf{f}_{\varepsilon^-,n+1}^{ext,e}; \mathbf{f}_{u,n+1}^{ext,e}]^T . \tag{A.20}$$

The element contributions to the internal force reads as:

$$\mathbf{f}_{\boldsymbol{\varepsilon}^w,n+1}^{int,e} =$$

$$\int \nabla \cdot \mathbf{N}_{\boldsymbol{\varepsilon}^w}^T \left[\frac{\mathbf{u}_{n+1}^h - \tilde{\mathbf{u}}_{n+1}^h}{\alpha \Delta t} + \frac{RT}{\alpha} \Phi^w \nabla \varepsilon^{w^h} + \frac{RT}{\alpha} \Phi^w \frac{c^+}{\varepsilon^{+^h}} \nabla \varepsilon^{+^h} + \frac{RT}{\alpha} \Phi^w \frac{c^-}{\varepsilon^{-^h}} \nabla \varepsilon^{-^h}\right]_{n+1} dV ,$$

$$\mathbf{f}_{\varepsilon^+,n+1}^{int,e} =$$

$$\int_\Omega \nabla \cdot \mathbf{N}_{\varepsilon^+}^T \left[-\frac{RT}{\alpha} \Phi^w c^F \nabla \cdot \mathbf{N}_{\boldsymbol{\varepsilon}^w} \varepsilon^w - \left(\frac{\Phi^w c^+ D^+}{\varepsilon^{+^h}} + \frac{RT}{\alpha} \Phi^w \frac{(c^+)^2}{\varepsilon^{+^h}} - \frac{RT}{\alpha} \Phi^w \frac{c^+ c^-}{\varepsilon^{+^h}}\right) \nabla \varepsilon^{+^h} + \left(\frac{\Phi^w c^- D^-}{\varepsilon^{-h}} + \frac{RT}{\alpha} \Phi^w \frac{(c^-)^2}{\varepsilon^{-h}} - \frac{RT}{\alpha} \Phi^w \frac{c^+ c^-}{\varepsilon^{-h}}\right) \nabla \varepsilon^{+^h}\right]_{n+1} dV,$$

(A.21)

$$\mathbf{f}_{\boldsymbol{\varepsilon}^-,n+1}^{int,e} =$$

$$\int_\Omega \nabla \cdot \mathbf{N}_{\boldsymbol{\varepsilon}^-}^T \left[-\frac{RT}{\alpha} \Phi^w c^F \nabla \cdot \mathbf{N}_{\boldsymbol{\varepsilon}^w} \varepsilon^w + \left(\frac{\Phi^w c^+ D^+}{\varepsilon^{+^h}} + \frac{RT}{\alpha} \Phi^w \frac{(c^+)^2}{\varepsilon^{+^h}} + \frac{RT}{\alpha} \Phi^w \frac{c^+ c^-}{\varepsilon^{+^h}}\right) \nabla \varepsilon^{+^h} +$$

$$\left(\frac{\Phi^w c^- D^-}{\varepsilon^{-h}}+\frac{RT}{\alpha}\Phi^w\frac{(c^-)^2}{\varepsilon^{-h}}+\frac{RT}{\alpha}\Phi^w\frac{c^+c^-}{\varepsilon^{-h}}\right)\nabla\varepsilon^{-h}\Big]_{n+1} dV +$$

$$\int \nabla\cdot\mathbf{N}^T_{\boldsymbol{\varepsilon}^-}\left(\Phi^w c^{k^h}\frac{\mathbf{u}^h_{n+1}-\tilde{\mathbf{u}}^h_{n+1}}{\alpha\Delta t}\right)dV+\int\mathbf{N}^T_{\boldsymbol{\varepsilon}^-}\left(\Phi^w\frac{c^{k^h}_{n+1}-\tilde{c}^{k^h}_{n+1}}{\alpha\Delta t}\right)dV,$$

$$\mathbf{f}^{int,e}_{\mathbf{u},n+1}=\int\mathbf{B}^T_{\mathbf{u}}\left[\mathbf{D}_{elas}\mathbf{H}\mathbf{u}^h-RT\varepsilon^{w^h}\mathbf{I}+RT\Phi c^{k^h}\mathbf{I}-B_w\mathbf{H}\mathbf{u}^h\right]_{n+1}dV,$$

where $\mathbf{D}_{elas}$ is the elastic constitutive matrix:

$$D_{elas}=\xi\begin{bmatrix}1-\nu & \nu & \nu & & & \\ \nu & 1-\nu & \nu & & \tilde{0} & \\ \nu & \nu & 1-\nu & & & \\ & & & \frac{1-2\nu}{2} & 0 & 0\\ & \tilde{0} & & 0 & \frac{1-2\nu}{2} & 0\\ & & & 0 & 0 & \frac{1-2\nu}{2}\end{bmatrix}, \quad (A.22)$$

with $\xi=\frac{E}{(1+\nu)(1-2\nu)}$.

In the case of the external force vector the element contributions have the following expressions:

$$\mathbf{f}^{ext,e}_{\mathbf{u},n+1}=\int\mathbf{N}^T_{\mathbf{u}}\boldsymbol{\sigma}^{*h}_{n+1}\,dV,$$

$$\mathbf{f}^{ext,e}_{\boldsymbol{\varepsilon}^w,n+1}=-\int\mathbf{N}^T_{\boldsymbol{\varepsilon}^{wh}}\mathbf{J}^{w*^h}_{n+1}\,dV, \quad (A.23)$$

$$\mathbf{f}^{ext,e}_{\boldsymbol{\varepsilon}^+,n+1}=\int\mathbf{N}^T_{\boldsymbol{\varepsilon}^+}(\mathbf{J}^{+*^h}_{n+1}-\mathbf{J}^{-*^h}_{n+1})\,dV,$$

$$\mathbf{f}^{ext,e}_{\boldsymbol{\varepsilon}^-,n+1}=-\int\mathbf{N}^T_{\boldsymbol{\varepsilon}^+}\left(\mathbf{J}^{+*^h}_{n+1}+\mathbf{J}^{-*^h}_{n+1}\right)dV.$$
